# Molecular Investigation in Early‐Onset Interstitial Lung Disease: Results From 699 Unrelated Patients

**DOI:** 10.1111/resp.70132

**Published:** 2025-10-03

**Authors:** Camille Louvrier, Nadia Nathan, Vincent Cottin, Tifenn Desroziers, Valérie Nau, Yohan Soreze, Florence Dastot‐Le Moal, Philippe Reix, Diane Bouvry, Caroline Thumerelle, Martine Reynaud‐Gaubert, Alice Hadchouel, Grégoire Prévot, Effrosyni Manali, Caroline Kannengiesser, Ibrahima Ba, Serge Amselem, Véronique Houdouin, Raphaël Borie, Marie Legendre, RespiRare and OrphaLung

**Affiliations:** ^1^ Medical Genetics Department Armand Trousseau Hospital, Sorbonne Université, Assistance Publique – Hôpitaux de Paris, Molecular Genetics Unit Paris France; ^2^ Sorbonne Université, Inserm UMR_S933, Laboratory of Childhood Genetic Diseases, Armand Trousseau Hospital Paris France; ^3^ Pediatric Pulmonology Department and Reference Center for Rare Lung Diseases RespiRare Armand Trousseau Hospital, Sorbonne Université, Assistance Publique – Hôpitaux de Paris Paris France; ^4^ Department of Respiratory Medicine National Reference Centre for Rare Pulmonary Diseases, Hospices Civils de Lyon, Université Lyon 1, UMR754, INRAE, ERN‐LUNG Lyon France; ^5^ Assistance Publique Hôpitaux de Paris, Intensive Care Unit, Armand Trousseau Hospital Paris France; ^6^ Pediatric Pulmonology Department Hôpital Femme Mère Enfant, Hospices Civils de Lyon Lyon France; ^7^ Service de Pneumologie et Oncologie Thoracique, Centre Constitutif Maladies Pulmonaires Rares de L'adulte, Assistance Publique‐Hôpitaux de Paris, Hôpital Avicenne Paris France; ^8^ Pediatric Pulmonology and Allergy Unit, Hôpital Jeanne de Flandre, University Lille, CHU Lille Lille France; ^9^ Department of Respiratory Medicine and Lung Transplantation Assistance Publique ‐ Hôpitaux de Marseille, Hôpital Nord; Aix‐Marseille Université Marseille France; ^10^ Assistance Publique – Hôpitaux de Paris, Hôpital Universitaire Necker‐Enfants Malades, Service de Pneumologie Pédiatrique, Centre de Référence Pour les Maladies Respiratoires Rares de L'enfant, INSERM U1151 INEM, Université Paris Cité Paris France; ^11^ Service de Pneumologie, Hôpital Larrey Toulouse France; ^12^ 2nd Pulmonary Department General University Hospital “Attikon”, Medical School, National and Kapodistrian University of Athens Athens Greece; ^13^ Genetics Department Bichat Hospital, AP‐HP, Université Paris Cité Paris France; ^14^ Assistance Publique Hôpitaux de Paris, Hôpital Universitaire Robert Debré, Service de Pneumologie Pédiatrique, Centre de Référence Pour les Maladies Respiratoires Rares de l'Enfant, INSERM U1151 INEM, Université Paris Cité Paris France; ^15^ Université Paris Cité, Inserm, PHERE, Hôpital Bichat, AP‐HP, Service de Pneumologie A, Centre Constitutif du Centre de Référence Des Maladies Pulmonaires Rares, FHU APO, LLO Paris France

**Keywords:** genetics, interstitial lung disease, pulmonary fibrosis, surfactant

## Abstract

**Background and Objective:**

Interstitial lung diseases (ILDs) are rare and severe respiratory conditions that may ultimately result in pulmonary fibrosis (PF). The objective of this study was to present the results of molecular diagnosis of early‐onset ILD (from neonates to young adults < 50 years) in a reference genetic diagnostic laboratory.

**Methods:**

DNAs from 699 index cases and 190 relatives were studied over 6 years by Sanger and/or targeted next generation sequencing of surfactant‐related genes and other genes involved in early‐onset ILD.

**Results:**

Pathogenic/likely pathogenic variants were evidenced for 62 patients (8.9%). The genes most frequently involved were *SFTPA2* (13/62), followed by *ABCA3* (12/62) and *SFTPC* (10/62). Among index cases for whom precise clinical data were available (*n* = 542), indications associated with a high molecular diagnostic yield were pulmonary alveolar proteinosis (61.5%, 8/13; *p* < 0.0007); family history of ILD/PF and lung cancer (36.8%, 7/19; *p* = 0.0132) and newborns > 32 weeks gestation with neonatal respiratory distress (14.8%, 9/61). The proportion of positive molecular investigations culminated in two age groups over the lifespan: 23.3% (7/30) in children aged 1 to 10 years, and 18.3% (15/82) in adults aged 30 to 40 years. Over the 6‐year period, 190 relatives were subjected to testing in order to perform segregation studies (*n* = 123) and/or predictive testing (*n* = 79).

**Conclusion:**

This study highlights the specific patient's characteristics associated with a high or low molecular diagnostic yield in clinical practice. Furthermore, it emphasises the importance of establishing a molecular diagnosis in order to provide genetic counselling to the family.

## Abstract 2

Interstitial lung diseases (ILDs) are rare and devastating respiratory diseases that can lead to pulmonary fibrosis (PF). This study summarises 6 years of molecular diagnosis of early‐onset ILD and highlights the main indications associated with a high or low molecular diagnostic yield.

**See related** editorial

## 1 Introduction

Interstitial lung disease (ILD)—also known as diffuse parenchymal lung disease—is an umbrella term for a broad spectrum of disorders of the distal lung parenchyma that can affect patients of all ages. These disorders, which are associated with high morbidity and mortality, are characterised by the presence of diffuse infiltrates on lung imaging and eventual progressive evolution towards pulmonary fibrosis (PF). Several dozen distinct entities of ILD have been described, ranging from ultra‐rare disorders to more common diseases such as idiopathic pulmonary fibrosis (IPF) [1]. IPF is a severe disease with an estimated incidence of 20 per 100,000 men and 13 per 100,000 women [2], affecting mainly older adults with a median survival of 3 to 5 years after clinical diagnosis [3]. Several classifications of ILD have been proposed and differ between children and adults [4]. Childhood interstitial lung disease (chILD) appears to be even rarer than adult ILD, with an estimated incidence of 4.4 per million children [5]. It can present from birth to adolescence with a wide range of severity, from pauci‐symptomatic to severe disease leading to pulmonary transplantation or death [6].

Many monogenic diseases can lead to ILD, with telomere‐related genes (TRG) and surfactant‐related genes (SRG) being the most commonly encountered [7]. In adult patients, pathogenic variants in TRG represent the main cause of Mendelian ILD irrespective of the age of onset, with 21% to 36% genetic tests positive in familial PF, depending on the studies, compared to 3% to 8% for SRG [8, 9, 10]. However, variants in SRG are more frequently encountered in adult patients with early‐onset ILD [9]. In chILD, a genetic cause is currently identified in approximately 20% of patients tested, and variants in SRG are the most frequently encountered [6]. Surfactant‐related genes (i.e., *SFTPA1*, *SFTPA2*, *SFTPB*, *SFTPC*, *ABCA3* and *NKX2‐1*) encode proteins involved in surfactant metabolism, a complex mixture of proteins (10%) and lipids (90%) that prevents collapse at the end of expiration by reducing surface tension at the air‐water interface of the lung alveoli. A number of other genes have been identified as playing a role in monogenic forms of chILD and early‐onset ILD. These include genes associated with pulmonary alveolar proteinosis (*MARS1*, *CSF2RA, CSF2RB, OAS1* and *GATA2*), autoinflammatory disorders (*STING1* and *COPA*) and other complex syndromes (*TBX4*, *FARSA*, *FARSB* and *FLNA*) [6, 8].

To date, studies of genetic forms of ILD have concentrated on either paediatric or adult populations. The aim of the present study is to present the results of molecular investigations in ILD and PF in patients from infancy to adulthood. To this end, we have evaluated the contribution of variants in surfactant‐related genes and other genes involved in early‐onset ILD by targeted next generation sequencing (NGS) in a large cohort of deeply phenotyped patients.

## 2 Patients and Methods

### 2.1 Patients and Samples

Blood DNA samples from 699 probands and 190 relatives were studied between 2018 and 2023. Patients were diagnosed by pulmonologists and paediatric pulmonologists mainly from French centres, but also from a Greek centre (24 patients). The main inclusion criterion was an age of respiratory symptoms onset before the age of 50 or, in the case of a family history, after 50 years but with at least one affected relative with onset before the age of 50. If age of onset was not specified, age at sampling was used to include the patient. The remaining patients over the age of 50, or those with a strong suspicion of dyskeratosis congenita, were not included in this study, but were tested with another targeted next generation sequencing (NGS) panel specifically targeting TRG and performed at another centre. These inclusion criteria are in line with the French diagnosis and care protocols (“Fibroses pulmonaires génétiques de l'adulte*”* https://www.has‐sante.fr/upload/docs/application/pdf/2022‐06/respifil_pnds_ag_fpg.pdf *and* “Pneumopathies interstitielles diffuses de l'enfant” https://www.has‐sante.fr/upload/docs/application/pdf/2017‐11/pneumopathies_interstitielles_diffuses_de_lenfant_‐_pnds.pdf) and the European Respiratory Society (ERS) statement. After information on genetic testing, written consent was obtained from all participants or their legal representatives by the primary physician in accordance with the French law on bioethics. For each patient, a standardised form was used to summarise the relevant clinical information, including the age of onset and any family history of respiratory disease (Supporting Information).

### 2.2 Genetic Studies

DNA was isolated from peripheral blood leukocytes using the Maxwell 16 system and the Maxwell 16 LEV Blood DNA Kit (Promega).

**FIGURE 1:**
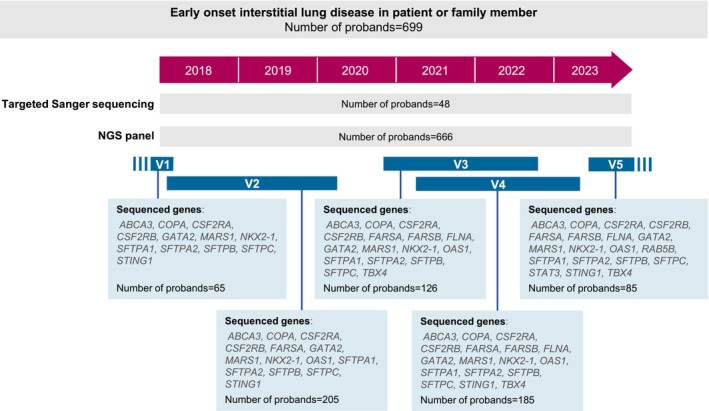
Molecular strategy used and number of unrelated probands analysed (*N* = 699). Timeline showing the number of patients sequenced by Sanger (*N* = 48) and/or by NGS (*N* = 666) and the versions of the NGS panels (V1 to V5). The sequenced genes are indicated for each version of the NGS panel. Fifteen patients underwent an initial targeted Sanger analysis followed by a second NGS analysis. Note that *STING1* was missing from version V3. This omission was quickly identified, after which a new version (V4) was purchased. During the V3 period, patients suspected of having SAVI (autoimmunity and/or extrapulmonary symptoms, such as arthralgia or skin involvement) were systematically sequenced using the V4 targeted panel.

Targeted NGS was performed on all the coding exons and the intronic flanking regions of the following list of genes: (i) *SFTPA1*, *SFTPA2*, *SFTPB*, *SFTPC, ABCA3, NKX2‐1, COPA, MARS1, CSF2RA, CSF2RB and GATA2* for all samples; (ii) *STING1*, *FARSA*, *FARSB*, *FLNA, OAS1, RAB5B, STAT3* and *TBX4* depending on the version of the targeted panel (Roche Sequencing, Pleasanton, CA) that evolved with time (Figure 1). NGS was conducted on a Miseq (Illumina, San Diego, CA) platform in accordance with the manufacturer's instructions. The sequence data were analysed by using an in‐house bioinformatics pipeline. The sequence reads in fastq format were aligned to the reference human genome (hg19) with BWA and Bowtie2. The variant calling was performed with GATK and VarScan with a threshold for the variant allele fraction (VAF) of 10%. Variant calls in VCF format were then annotated through Annovar. Copy number variants were analysed using mean coverage depth of the capture regions (https://gitlab.inria.fr/HCL/decova/).

Sanger sequencing was conducted on an ABI 3730XL automated capillary DNA sequencer (Applied Biosystems, Foster City, CA) with a specific focus on an exon or a gene. Sequencing data were then analysed using the Seqscape software.

The identified variants were classified in accordance with the classification of the American College of Medical Genetics and Genomics (ACMG) and included pathogenic (P), likely pathogenic (LP), variant of uncertain significance (VUS), likely benign (LB), and benign (B) [11].

### 2.3 Data Analysis

Continuous variables were expressed as mean. Categorical variables were expressed as *n* (%). The statistical analyses were performed with the Prism software (GraphPad Software, San Diego, CA): two‐sided Fisher's exact test, and adjusted *p* values for multiple comparison correction were calculated using the Bonferroni method.

## 3 Results

### 3.1 Cohort of Patients

A total of 699 unrelated patients were analysed in this study, mainly by sequencing of a custom NGS panel (*n* = 666) and/or by targeted Sanger sequencing (*n* = 48). Fifteen patients underwent an initial targeted Sanger analysis followed by a second NGS analysis, mainly in an emergency context, to enable a rapid initial assessment (Figure 1). The male‐to‐female ratio was 1.4 (405 males, 294 females). The mean age of onset was 27 years (median 32 years), with a wide range from birth to age 79.

The number of patients per age group between birth and over 50 years of age is provided in Table 1. For 157 patients, the age of disease onset was not specified on the clinical standardised form. These patients were retained to establish the overall diagnostic yield but removed for all studies by category (age of disease onset, indications and family history). The results including these 157 patients are shown in Figure S1. A large proportion of patients, 30.3% (164/542), were diagnosed with ILD prior to the age of one, and only 14.2% (77/542) after the age of 50. A total of 218 patients were diagnosed at or below the age of 18 (120 males, 98 females), while 324 patients were diagnosed above the age of 18 (195 males, 129 females).

**TABLE 1:** Cohort summary by age of onset, indication, family history and molecular diagnoses.

	Total (*n* = 542) (%)	Positive (*n* = 60) (%)	Diagnostic yield (11.1%)	Adjusted *p* value
Age of onset (years)				
Age ≤ 1	164 (30.3%)	21 (35%)	12.8%	1
1 < age ≤ 10	30 (5.5%)	7 (8.3%)	23.3%	0.2149
10 < age ≤ 18	24 (4.4%)	1 (1.7%)	4.2%	1
18 < age ≤ 30	46 (8.5%)	6 (10%)	13.0%	1
30 < age ≤ 40	82 (15.1%)	15 (25%)	18.3%	0.252
40 < age ≤ 50	119 (22.0%)	10 (16.7%)	8.4%	1
Age > 50	77 (14.2%)	0 (0%)	0%	**0.0063**
Indication				
Neonatal RDS < 32 WG	9 (1.7%)	0 (0%)	0%	1
Neonatal RDS ≥ 32 WG	61 (11.3%)	9 (15%)	14.8%	1
ILD/PF	410 (75.6%)	37 (61.7%)	9.0%	1
ILD/PF + Lung cancer	8 (1.5%)	3 (5%)	37.5%	0.2821
PAP	13 (2.4%)	8 (13.3%)	61.5%	**< 0.0007**
Alveolar haemorrhage	17 (3.1%)	1 (1.7%)	5.9%	1
Others	24 (4.4%)	2 (3.3%)	8.3%	1
Family history				
No family history	279 (51.5%)	29 (48.3%)	10.4%	1
ILD/PF	87 (16.1%)	6 (10%)	6.9%	1
Lung cancer	29 (5.3%)	4 (6.7%)	13.8%	1
ILD/PF + Lung cancer	19 (3.5%)	7 (11.7%)	36.8%	**0.0132**
Undefined respiratory disease	32 (5.9%)	3 (5%)	9.4%	1
Data not available	96 (17.7%)	11 (18.3%)	11.4%	1

*Note*: A two‐sided Fisher's exact test was used with multiple comparison correction (adjusted *p* value) and a *p* < 0.05 (in bold) indicates that the molecular diagnostic yield is significantly different from the overall molecular diagnostic yield.

Abbreviations: ILD, interstitial lung diseases; Others, other respiratory diseases such as cystic disease, obliterative bronchiolitis or emphysema; PAP, pulmonary alveolar proteinosis; PF, pulmonary fibrosis; RDS, respiratory distress syndrome; WG, week's gestation.

The majority of patients, 75.6% (410/542), had a clinical diagnosis of ILD or PF without lung cancer. The remaining patients presented persistent neonatal respiratory distress syndrome (RDS) (12.9%, 70/542), of whom 9 were born extremely or very preterm (less than 32 weeks of gestation, WG), diffuse alveolar haemorrhage (3.1%, 17/542), ILD or PF and lung cancer (1.5%, 8/542), ILD related to pulmonary alveolar proteinosis (PAP) (2.4%, 13/542) and other respiratory diseases such as cystic disease, obliterative bronchiolitis, or emphysema (24/542) (Table 1). A family history of respiratory disease was reported in 30.8% (167/542) of patients. Among them, 87/167 (52.1%) reported a familial form of ILD or PF without lung cancer, and 19/167 (11.4%) an aggregation of ILD or PF and lung cancer. The remaining family backgrounds included the presence of lung cancer without ILD or PF (29/167) and undefined respiratory diseases (32/167) (Table 1).

### 3.2 Molecular Diagnostic Yield

**FIGURE 2:**
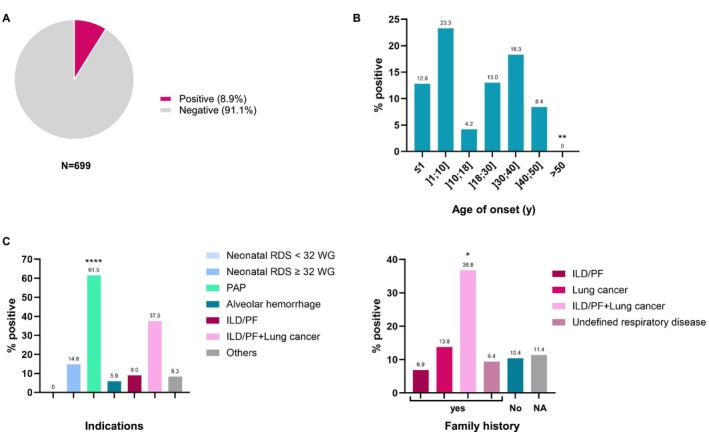
Analysis of the molecular diagnostic yield in unrelated probands. (A) Overall molecular diagnostic yield. A positive molecular diagnosis corresponds to an unambiguous study including one (dominant) or two (recessive) pathogenic/likely pathogenic variants. (B–D) Distribution of the % of positive diagnoses—for the 542 unrelated probands for whom precise clinical data were available—by age of disease onset (B), by clinical diagnosis of tested probands (C) and by family history (D). Figure S1 provides the results for the 699 unrelated probands. ILD, interstitial lung disease; NA, not available; obliterative bronchiolitis, or emphysema; Others, other respiratory diseases such as cystic disease; PAP, pulmonary alveolar proteinosis; PF, pulmonary fibrosis; RDS, respiratory distress syndrome. Fisher's exact tests with multiple comparison corrections (adjusted *p* value) were performed to compare each % positive group to the overall % diagnosis (*: *p* < 0.05; **: *p* < 0.01; ****: *p* < 0.0001).

Among the 699 unrelated probands sequenced and analysed during the study period, a monogenic cause of ILD was determined in 62 patients (14 by Sanger sequencing and 48 by NGS panel) with unambiguous molecular diagnosis (likely pathogenic (LP) and pathogenic (P) variants), corresponding to a positive rate of 8.9% (Figure 2A, Table S1). The positive rate was found to vary according to the age of onset, the indication for genetic study, and the family history of respiratory diseases. To perform the statistical analyses per group as accurately as possible, the 157 patients for whom information about the age of disease onset was lacking were excluded from the main results (Table 1, Figure 2B,C). However, the results including these patients are available in Figure S1, and, while less precise, show a similar trend. After removing these 157 patients, the overall molecular diagnostic yield reached 11.1% (60/542).

With regard to the age of onset, 48.3% (29/60) of molecular diagnoses were reported in children aged 18 or under, while 51.7% (31/60) were observed in adult patients aged over 18 (Figure 2B).

The highest positive rates were observed for the patient age group [1;10] with 23.3% (7/30) positive and the group [30;40] with 18.3% (15/82) positive. Conversely, no molecular diagnosis was made for patients with an age of onset above 50 years (0/77; *p* = 0.0063).

In terms of clinical phenotype, the positive rate in patients with ILD/PF was 9.0% (37/410), irrespective of the age of onset (Figure 2C). In the group of patients with neonatal RDS in infants born after 32 WG, this positive rate was 14.8% (9/61), but dropped to 0.0% (0/9) in extremely or very preterm (< 32 weeks) infants. In the case of less common indications, several points require further consideration. (i) The molecular diagnostic yield was particularly high in patients with PAP, with positive molecular diagnostics obtained for 61.5% (8/13; *p* = < 0.0007) of patients. (ii) The association of ILD/PF with lung cancer also had a high positive rate of 37.5% (3/8). (iii) Conversely, the positive rate was low for patients with diffuse alveolar haemorrhage (5.9%; 1/17) and other respiratory diseases (8.3%; 2/24) (Figure 2C, Table 1).

Furthermore, the prevalence of positive cases was examined in relation to the presence or absence of a family history of respiratory disease (Figure 2D, Table 1). No difference was observed between the three groups: no family history (10.4%; 29/279), family history (12.0%; 20/167) and data not available (11.4%; 11/96). Nevertheless, a closer examination revealed that the positive rate was notably elevated in cases of a family history of ILD/PF and lung cancer (36.8%; 7/19; *p* = 0.0132). Conversely, the presence of another case of ILD/PF in the family (without lung cancer) was not found to be associated with a higher positive rate (6.9%; 6/87) (Figure 2D, Table 1).

### 3.3 Main Genes Involved in Early‐Onset ILD

**FIGURE 3:**
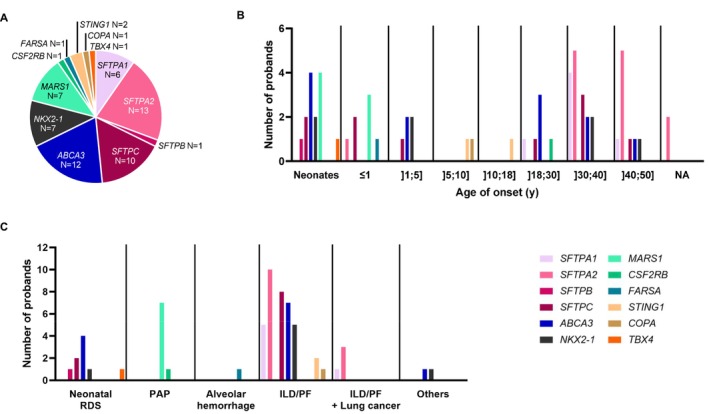
Distribution of the involved genes in the molecular diagnoses from the cohort of 699 unrelated probands. (A) Number of positive cases per gene. (B) Distribution of the involved genes by age of onset and (C) indication of the molecular study.

A total of 62 positive molecular diagnostics were found in 62 of the overall 699 unrelated patients during the course of this study, involving 12 different genes (*SFTPA1, SFTPA2*, *SFTPB*, *SFTPC*, *ABCA3*, *NKX2‐1*, *MARS1*, *CSF2RB, FARSA, STING1*, *COPA* and *TBX4*) and 59 different LP/P variants. The principal genes implicated were *SFTPA2* (*n* = 13), *ABCA3* (*n* = 12) and *SFTPC* (*n* = 10) (Figure 3A, Table S1).

The distribution of the genes involved displayed notable differences according to the age of onset and indication. In neonates, the most frequently involved genes were *ABCA3* and *MARS1*. The *SFTPA1* and *SFTPA2* genes were almost exclusively identified in adult patients between the ages of 30 and 50 years. *ABCA3, SFTPC*, *and NKX2‐1* variants were identified in patients with heterogeneous phenotypes, from neonatal forms to adult patients (range 0–50 years) (Figure 3B, Table S1).

Neonatal RDS and ILD/PF were found to exhibit a genetic heterogeneity. *ABCA3* was identified as the primary contributor to neonatal RDS, but *SFTPB*, *SFTPC*, *NKX2‐1* and *TBX4* were also involved. Seven different genes (*SFTPA1*, *SFTPA2*, *SFTPC*, *ABCA3*, *NKX2*‐1, *STING1* and *COPA*) corresponding to the molecular diagnosis of 39 patients were involved in this study for a clinical presentation of isolated or non‐isolated ILD/PF. Conversely, the molecular diagnosis of PAP was associated with bi‐allelic *MARS1* variants in seven out of eight patients. In instances where an association with ILD/PF and lung cancer was reported, all patients with a positive molecular diagnosis exhibited *SFTPA1* or *SFTPA2* variants (*n* = 4) (Figure 3C, Table S1).

### 3.4 Impact of Molecular Diagnosis for Families

**FIGURE 4:**
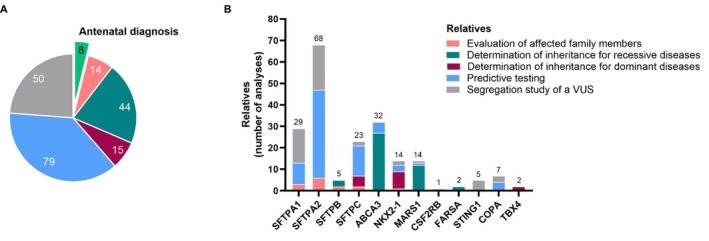
Genetic counselling. (A) Number of antenatal diagnoses and studies of relatives according to screening indications during the 6‐year study period. (B) Distribution of analysed genes in relatives, according to screening indications.

In order to evaluate the utility of molecular diagnosis for genetic counselling, we conducted an analysis of the number of prenatal diagnoses and genetic testing of relatives over the course of the study period (2018–2023) (Figure 4). A total of eight antenatal diagnoses were carried out for *ABCA3* (*n* = 6), *SFTPC* (*n* = 1) and *SFTPA2* (*n* = 1). With regard to relatives, 190 distinct family members underwent genetic testing (Figure 4A). The indications for screening relatives were diverse and included: (i) evaluation of affected family members (*n* = 14); (ii) determination of inheritance of bi‐allelic variants (recessive disease) by parent testing (*n* = 44); (iii) determination of inheritance or de novo status of a heterozygous variant (dominant disease) by parent testing (*n* = 15); (iv) predictive testing of an asymptomatic family member (*n* = 79) and (v) finally, a segregation study could also be done in relatives to assess a VUS (*n* = 50), with potential to reclassify it as LB or LP. Genetic testing of relatives was performed for 12 genes (Figure 4B). Of the 190 tested relatives subjected to molecular diagnosis, approximately half were tested for *SFTPA2* (*n* = 68 including 6 evaluation of affected relatives, 41 predictive testing and 21 segregation studies to assess a VUS) and *SFTPA1* (*n* = 29 including 3 evaluation of affected relatives, 10 predictive testing and 16 segregation studies to assess a VUS).

## 4 Discussion

This study presents the findings of 6 years (2018–2023) of molecular investigations conducted at a French reference laboratory for the molecular diagnosis of ILD. The molecular diagnostic yield reached 8.9% across all age groups and clinical contexts. It is important to note that the overall result masks significant differences depending on the indication of the genetic study and the age group. Furthermore, the study highlights the crucial role of prenatal and pre‐symptomatic screening in affected families.

A precise selection of patients based on their phenotype has the potential to markedly enhance the positive rate. The following indications appear to be associated with a high molecular diagnostic yield: PAP without autoantibodies, ILD/PF with lung cancer diagnosed before the age of 50, and neonatal RDS in full‐term (or late preterm) infants.

Among the 542 patients with a precise age of disease onset, neonatal RDS in infants born ≥ 32 WG is characterised by a better molecular diagnostic yield (14.8%). In these patients, the molecular diagnoses ranged from surfactant disorders to developmental disorders, often confirmed by lung histology [12]. The prognosis of such conditions is extremely severe, with the majority of cases resulting in death during the neonatal period. In cases of such dramatic presentation, molecular diagnosis can contribute to the optimal management and genetic counselling of the parents. Pulmonary alveolar proteinosis (PAP) is a rare form of chronic interstitial lung disease characterised by the intra‐alveolar accumulation of lipoproteinaceous material. PAP can be autoimmune, secondary and genetic. Whereas autoimmune PAP is the main cause in adults, genetic defects account for a large proportion of cases in infants and children [13, 14]. The present study established a definitive molecular diagnosis in 61.5% of PAP cases, primarily among neonates. Among the patients with a positive molecular diagnosis, 7/8 carried bi‐allelic variants in *MARS1* and four were homozygous for the same c.1700C>T p.(Ser567Leu) pathogenic variant (Table S1). This specific genotype is the result of a founder effect that is prevalent on the French island La Reunion, where the incidence of PAP is at least 1 in 10,000 newborns [15, 16], and therefore explains the high diagnostic yield of PAP in our French cohort. As patients with this disease now benefit from targeted therapy with methionine substitution [17], early molecular diagnosis is essential to improve their prognosis.

Apart from newborns, the age of disease onset represents a significant criterion, exhibiting two principal peaks in terms of positive rates. The first peak is observed during infancy (1–10 years), with a positive rate of 23.3%. The second peak is observed in young adult patients (30–40 years), with a positive rate of 18.3%. The high molecular diagnostic yield in this first group was expected, given that chILD related to surfactant disorders is typically symptomatic during the first years of life [6]. The proportion in the young adult group is more surprising and reflects the very early onset of ILD/PF associated with SRG in adults compared to the age of onset of ILD/PF related to TRG [18]. This was also suggested by van Moorsel et al., who reported in a retrospective study a median age of onset of 45 years in adult patients carrying LP/P variants in *SFTPA1*, *SFTPA2* and *SFTPC* genes versus 62 years in adult patients with TRG LP/P variants [9]. In the present study, no positive molecular diagnosis was made in patients aged over 50 years (median age of the group: 57 years) at the onset of disease manifestation, despite the fact that the majority of them (64/77) had a family history of the disease. Therefore, the French recommendations to first analyse TRG in patients who develop ILD/PF over the age of 50 appear to be consistent with our findings.

Finally, the presence of a family history did not emerge as a highly predictive factor for a positive molecular diagnosis (diagnostic yield of 12.0% versus 10.4% in the family history group and no family history group respectively). This unexpected result, previously reported with TRG in patients referred for lung transplantation [19], may be explained by a number of factors. Firstly, the present study was based on molecular investigations of index cases, and for autosomal recessive diseases (e.g., *ABCA3*, *MARS1*, *CSF2RB*), most often concerned the first affected individual in the family. Furthermore, in infants, severe cases were frequently associated with *de novo* heterozygous variants for diseases that are transmitted in an autosomal dominant manner (e.g., *SFTPC*, *NKX2‐1*). Secondly, the combination of environmental exposures and genetic predispositions confers a multidimensional aetiology to adult ILD/PF and may have reduced the weight of family history as a positive predictive factor in our study [20]. In adults, environmental exposures such as cigarette smoking, as well as the common *MUC5B* promoter variant rs35705950, are significant confounding risk factors for ILD/PF [21, 22]. Of particular interest, however, was the high positive rate observed in cases with a family history of ILD/PF and lung cancer (36.8%; *p* = 0.0132). Although PF is known to increase the risk of lung cancer, this association should strongly recommend a molecular screening for SRG, especially *SFTPA1* and *SFTPA2*, which have recently been shown to be associated with a high risk of lung cancer, particularly in smokers [23].

The study also demonstrated the molecular heterogeneity associated with childhood or early‐onset ILD/PF. Of the 62 positive molecular diagnostics, 12 different genes were involved and 59 different LP/P variants, making the targeted NGS strategy relevant. Some genes were involved in only one patient in the cohort (*COPA*, *TBX4*, *FARSA* and *CSF2RB*), either because the disease is extremely rare (*FARSA* and *CSF2RB*) or because they may represent a differential diagnosis of typical ILD/PF (*TBX4*). The case of *COPA* is particularly noteworthy, as a pathogenic variant was identified in only one child with ILD and joint involvement, but over the study period, in no patient with diffuse alveolar haemorrhage (*n* = 17) contrary to expectations [24].

In summary, this study presents a comprehensive overview of several years of molecular diagnosis of early‐onset ILD in a reference genetic diagnostic laboratory. The study highlights very good indications for molecular testing (e.g., ILD and lung cancer, PAP) and indications associated with very poor molecular diagnostic yields (e.g., diffuse alveolar haemorrhage). The relatively low rate of positive results is likely attributable to both the context in which patients are included (i.e., newborns with RDS for whom we aim to exclude the hypothesis of a severe genetic disease, and young adults whose fibrosis may be attributed to multiple causes) and also the existence of genetic causes that remain to be identified. The study of negative patients by whole genome sequencing, as currently carried out in France within the framework of the *Plan France Médecine Génomique 2025* [25], will likely provide insight into this question. Indeed, this question remains topical in a context where, as this study illustrates, genetic counselling is of paramount importance.

## Author Contributions

**Camille Louvrier:** conceptualization (equal), data curation (lead), formal analysis (lead), investigation (lead), methodology (lead), validation (equal), writing – original draft (lead), writing – review and editing (lead). **Nadia Nathan:** conceptualization (equal), data curation (equal), formal analysis (equal), investigation (equal), validation (equal), writing – original draft (equal), writing – review and editing (equal). **Vincent Cottin:** data curation (supporting), writing – review and editing (supporting). **Tifenn Desroziers:** data curation (supporting), writing – review and editing (supporting). **Valérie Nau:** data curation (supporting), investigation (supporting), writing – review and editing (supporting). **Yohan Soreze:** data curation (supporting), writing – review and editing (supporting). **Florence Dastot‐Le Moal:** data curation (supporting), investigation (supporting), writing – review and editing (supporting). **Philippe Reix:** data curation (supporting), writing – review and editing (supporting). **Diane Bouvry:** data curation (supporting), writing – review and editing (supporting). **Caroline Thumerelle:** data curation (supporting), writing – review and editing (supporting). **Martine Reynaud‐Gaubert:** data curation (supporting), writing – review and editing (supporting). **Alice Hadchouel:** data curation (supporting), writing – review and editing (supporting). **Grégoire Prévot:** data curation (supporting), writing – review and editing (supporting). **Effrosyni Manali:** data curation (supporting), writing – review and editing (supporting). **Caroline Kannengiesser:** investigation (supporting), methodology (supporting), writing – review and editing (supporting). **Ibrahima Ba:** investigation (supporting), writing – review and editing (supporting). **Serge Amselem:** data curation (supporting), writing – review and editing (supporting). **Véronique Houdouin:** data curation (supporting), writing – review and editing (supporting). **Raphaël Borie:** data curation (supporting), investigation (supporting), methodology (supporting), writing – review and editing (supporting). **Marie Legendre:** data curation (equal), investigation (equal), methodology (supporting), validation (supporting), writing – review and editing (supporting).

## Ethics Statement

The study conforms to the Declaration of Helsinki regarding ethical principles for medical research. The study is approved by the Institutional Review Board (*Comité de protection des personnes* no. 20130604). Written informed consent for genetic testing was obtained from all participants or their legal representatives, in accordance with the French law on bioethics.

## Conflicts of Interest

Nadia Nathan reported grants or contracts from CORTICONEHI: Clinical trial, Million Dollar Bike Ride project for Neuroendocrine Cell Hyperplasia of Infancy, Chancellerie des Universités: Legs Poix and RespiFIL. Vincent Cottin reported consulting fees from Abbvie, Astra Zeneca, Avalyn, Boehringer Ingelheim, Celgene/BMS, CSL (Behring, Vifor), Ferrer/United Therapeutics, Gossamer, GSK, Liquidia, Pliant, PureTech, Roche, Roivant, Sanofi and Shionogi; payment or honoraria for lectures, presentations, speakers bureaus, manuscript writing or educational events by Boehringer Ingelheim, Ferrer/United Therapeutics, Roche and Sanofi; support for attending meetings and/or travel by Boehringer Ingelheim and Sanofi; participation on a Data Safety Monitoring Board or Advisory Board by GSK and Molecure; and leadership or fiduciary role in other board, society, committee or advocacy group, paid or unpaid by Fibrogen. Tifenn Desroziers reported grants or contracts from RespiFIL and from the COST Innovators Grant CIG16125 2022. Yohan Soreze reported grants or contracts from GFRUP and SFN. Philippe Reix reported a leadership or fiduciary role in other board, society, committee or advocacy group, paid or unpaid for the European Medicines Agency. Effrosyni D. Manali reported payment or honoraria for lectures, presentations, speakers bureaus, manuscript writing or educational events by Boehringer Ingelheim, DEMO Hellas and ELPEN Hellas, and support for attending meetings and/or travel by Boehringer Ingelheim and ELPEN Hellas. Raphaël Borie reported consulting fees from Boehringer Ingelheim, Ferrer and Sanofi, payment or honoraria for lectures, presentations, speakers bureaus, manuscript writing or educational events by Boehringer Ingelheim, and support for attending meetings and/or travel by Boehringer Ingelheim. The other authors have no conflicts of interest to report.

## Supporting information


**Figure S1:** Analysis of the molecular diagnostic yield in unrelated probands incorporating the 157 patients with no details concerning the age of disease onset. A. Distribution of the age of disease onset. B. Distribution of the % of positive diagnoses by age of disease onset, by clinical diagnosis of tested probands and by family history. PAP: pulmonary alveolar proteinosis, ILD: interstitial lung disease, PF: pulmonary fibrosis, RDS: respiratory distress syndrome, NA: not available. Fisher's exact tests with multiple comparison corrections (adjusted *p* value) were performed to compare each % positive groups to the overall % diagnosis (*: *p* < 0.0001).

**Data S1:** Supporting Information.

**Table S1:** Patients with a positive molecular diagnosis.

## Data Availability

The data that support the findings of this study are available from the corresponding author upon reasonable request.
