# Supplementary material for: Molecular Investigation in Early‐Onset Interstitial Lung Disease: Results From 699 Unrelated Patients
Source: Respirology. 2025 Oct 3;31(1):53–61. doi: 10.1111/resp.70132 (PMC12783964; doi:10.1111/resp.70132)
Supplement: Supplementary file 1 — Figure S1: Analysis of the molecular diagnostic yield in unrelated probands incorporating the 157 patients with no details concerning the age of disease onset. A. Distribution of the age of disease onset. B. Distribution of the % of positive diagnoses by age of disease onset, by clinical diagnosis of tested probands and by family history. PAP: pulmonary alveolar proteinosis, ILD: interstitial lung disease, PF: pulmonary fibrosis, RDS: respiratory distress syndrome, NA: not available. Fisher's exact tests with multiple comparison corrections (adjusted p value) were performed to compare each % positive groups to the overall % diagnosis (*: p < 0.0001). [file RESP-31-53-s003.pdf]

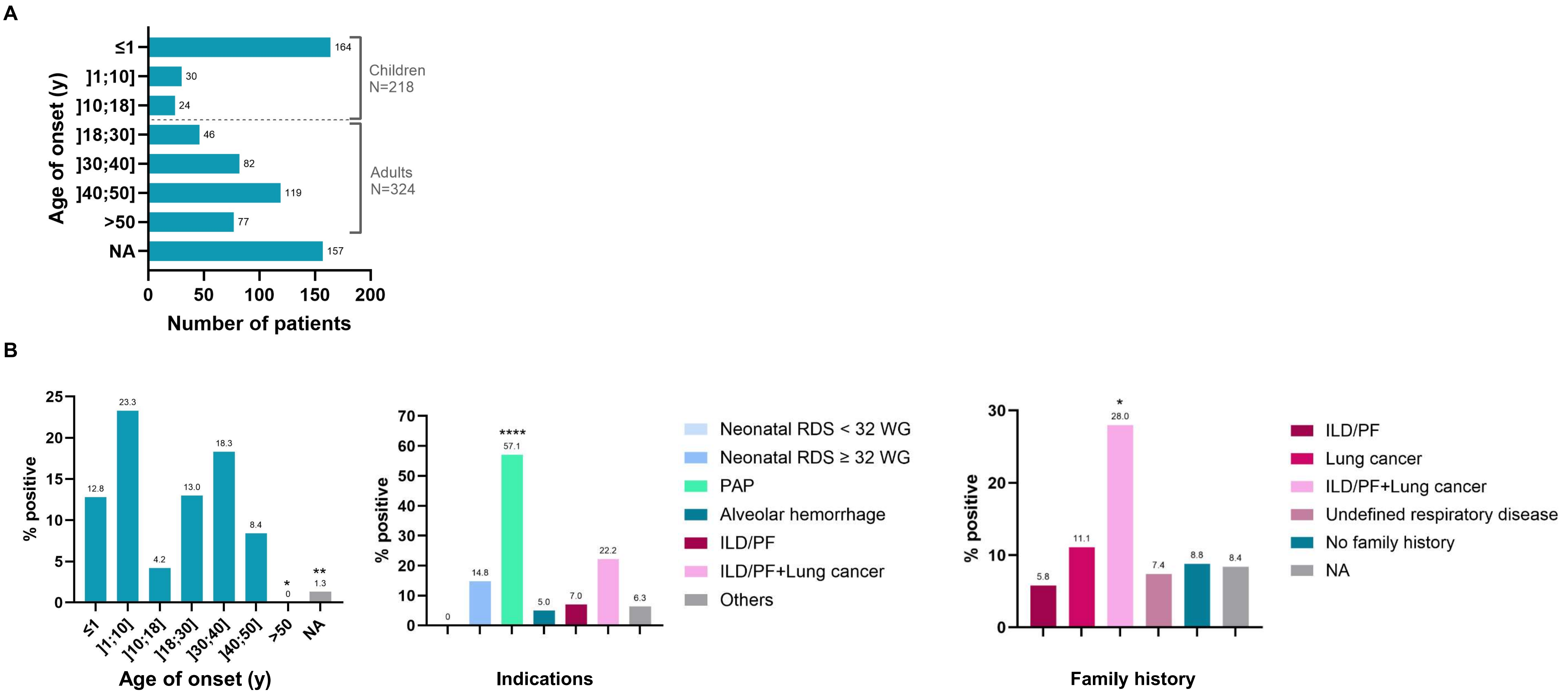

Figure S1. Analysis of the molecular diagnostic yield in unrelated probands incorporating the 157 patients with no details concerning the age of disease onset. A. Distribution of the age of disease onset. **B.** Distribution of the % of positive diagnoses by age of disease onset, by clinical diagnosis of tested probands and by family history. PAP: pulmonary alveolar proteinosis, ILD: interstitial lung disease, PF: pulmonary fibrosis, RDS: respiratory distress syndrome, NA: not available. Fisher's exact tests with multiple comparison corrections (adjusted p value) were performed to compare each % positive groups to the overall % diagnosis (*: p<0.05; **: p<0.01; **** p<0.0001).
