# Supplementary material for: Molecular Investigation in Early‐Onset Interstitial Lung Disease: Results From 699 Unrelated Patients
Source: Respirology. 2025 Oct 3;31(1):53–61. doi: 10.1111/resp.70132 (PMC12783964; doi:10.1111/resp.70132)
Supplement: Supplementary file 2 — Data S1: Supporting Information. [file RESP-31-53-s002.pdf]

GHU AP-HP.Sorbonne Université
DMU BioGEMH (Pr Lévy) - LBM de l'Est Parisien (Dr Vaubourdolle)
Département de Génétique Médicale - Pr. E. Le Guern

UF de Génétique Moléculaire – Pr. M. Legendre
Hôpital Armand Trousseau
Bâtiment Ketty Schwartz, porte B
26, rue du Dr Arnold Netter – 75571 PARIS Cedex 12

AP-HP. Sorbonne Université

Secrétariat : Tél. : + 33 (0)1 44 73 52 95 Fax : + 33 (0)1 44 73 52 19
secret.genetiquemoleculaire@aphp.fr

Dr. Camille Louvrier +33 (0)1 87 89 27 84 camille.louvrier@aphp.fr

Etude génétique des pneumopathies interstitielles diffuses (PID) et fibroses pulmonaires familiales et/ou à début précoce (Hors gènes du complexe télomérase)

Remplir une fiche par sujet prélevé et joindre le compte-rendu de RCP
Joindre un consentement ou une attestation de recueil de consentement par sujet prélevé

ETIQUETTE SUJET PRELEVE*

Individu prélevé : **atteint** ❑ **non atteint** ❑

Date de prélèvement :
Nom du préleveur :

Médecin référent senior :
Numéro APH ou RPPS* :
Service, Hôpital
N° de téléphone :
Email :

DIAGNOSTIC (Etiologie) :

Age ou date au début des manifestations respiratoires :
Forme familiale oui ❑ non ❑ ND ❑
Si oui préciser sur l'arbre au verso
Origines géographiques (père / mère) : /
Consanguinité parentale : oui ❑ non ❑ ND ❑

INDICATION

En dehors de ces indications, sauf accord préalable du laboratoire, le prélèvement ne sera pas techniqué

ENFANTS

- PID idiopathique ❑
- Détresse respiratoire néonatale à terme persistante ❑ *(préciser si avec ou sans HTAP)*
- Protéinose alvéolaire au LBA / biopsie ❑
- Hémorragie alvéolaire au LBA / biopsie ❑
- Biopsie pulmonaire compatible avec une PID ou une anomalie de développement pulmonaire ❑

ADULTES

PID fibrosante ou fibrose pulmonaire idiopathique avec
- Moins de 50 ans au diagnostic de la PID ❑
- Forme familiale de PID +/- cancer broncho-pulmonaire avec début avant 50 ans chez au moins l'un des apparentés ❑

Pour les adultes, préciser le résultat de l'analyse génétique des gènes du complexe télomérase :
Résultat : En cours ❑

ANTECEDENTS PERSONNELS

Détresse respiratoire néonatale oui ❑ non ❑
Prématurité, *Préciser le terme :* oui ❑ non ❑

Cancer broncho-pulmonaire oui ❑ non ❑
Préciser le type :

Autres antécédents

Arthralgies	oui ❑	non ❑
Polyarthrite rhumatoïde	oui ❑	non ❑
Auto-immunité / Dysimmunité	oui ❑	non ❑
Cirrhose hépatique, cholestase	oui ❑	non ❑
Canitie précoce, dystrophie unguéale	oui ❑	non ❑
Livedo, télangiectasies	oui ❑	non ❑
Chorée, hypotonie, mvts anormaux	oui ❑	non ❑
Hypothyroïdie périphérique	oui ❑	non ❑
Thrombopénie, anémie, myélodysplasie	oui ❑	non ❑

Si « oui » à au moins une réponse ou si autre atteinte, préciser :

Tabagisme (PA) oui ❑ non ❑
Exposition professionnelle oui ❑ non ❑
Préciser :

ELEMENTS ACTUELS

Oxygénothérapie oui ❑ non ❑
Transplantation pulmonaire oui ❑ non ❑ sur liste ❑

Scanner thoracique (joindre une coupe de TDM ou à défaut la radio de thorax initiale pour les nouveau-nés)
Préciser :

Biopsie pulmonaire oui ❑ non ❑
Résultat :

HISTOIRE FAMILIALE ET ARBRE GENEALOGIQUE

COMPLETER L'ARBRE GENEALOGIQUE

PID ou fibrose pulmonaire chez un autre membre de la famille oui ❑ non ❑

Si oui préciser l'individu sur l'arbre, son âge de début de la maladie et le type

Cancer bronchopulmonaire chez un autre membre de la famille oui ❑ non ❑

Si oui préciser l'individu sur l'arbre et le type

Autres antécédents familiaux : *préciser sur l'arbre généalogique*

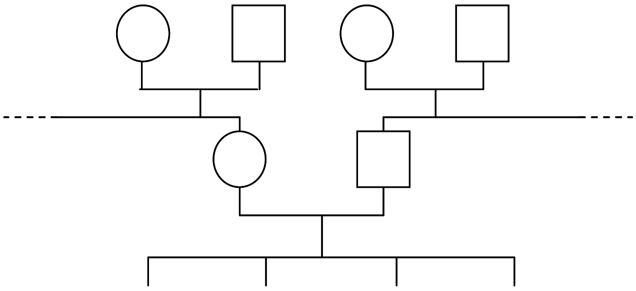

○ sujet féminin □ sujet masculin
● ■ sujet atteint, ○ □ sujet sain
flèche : sujet prélevé

MODALITES DE PRELEVEMENT ET D'EXPEDITION
(la réception avant le vendredi 12h est préférable)

Les prélèvements doivent être adressés au laboratoire avec cette fiche de renseignements
1 tube E.D.T.A. de 5 ml pour les adultes ou 1 tube de 3 ml pour les enfants, prélevés dans des conditions stériles et soigneusement agités par retournements.
Étiqueter chacun des tubes, avec les nom, prénom et date de naissance.
Conservation à température ambiante avec un acheminement si possible dans les 72 heures

Expédition du prélèvement par courrier :
Conditionner les tubes dans une boîte rigide étanche tapissée par un matériau absorbant en les protégeant les uns des autres.
Adresser par courrier rapide à température ambiante à l'adresse indiquée en en-tête.

ATTENTION
Joindre impérativement à tout prélèvement sanguin **le consentement éclairé du patient** et **un bon de commande** si hors AP-HP
