# Supplementary material for: Molecular Investigation in Early‐Onset Interstitial Lung Disease: Results From 699 Unrelated Patients
Source: Respirology. 2025 Oct 3;31(1):53–61. doi: 10.1111/resp.70132 (PMC12783964; doi:10.1111/resp.70132)
Supplement: Supplementary file 3 — Table S1: Patients with a positive molecular diagnosis. [file RESP-31-53-s001.pdf]

Table S1: Patients with a positive molecular diagnosis

Patient ID	Variation(s)	ACMG classification	Previous report of the patient	First references	Clinical phenotype	Age of onset (year)	Family history
SFTPA1 **(NM_005411), AD**							
18GM00745	c.556T>C, p.(Tyr186His) +/-	LP	(1)		PF	40	Yes (2 brothers with PF, died at ages 39 and 40)
19GM00179	c.631T>C p.(Trp211Arg) +/-	P		(2)	ILD	37	No
19GM00803	c.513T>G p.(Asn171Lys) +/-	LP	(1)		PF	50	Yes (Son: PF and lung adenocarcinoma, death at 39 years)
19GM00878	c.692G>A p.(Gly231Glu) +/-	LP	(1)		PF	48	No
8820GM001002	c.673G>A p.(Val225Met) +/-	P	(1)	(3)	Fibrosing ILD, death post-transplantation	20	Yes (PF and rheumatic arthritis in the mother, ILD and rheumatic arthritis in the sister, ILD in the brother, lung cancer in the maternal uncle)
8821GM000892	c.557A>C, p.(Tyr186Ser)	LP	(1)		PF, lepidic lung cancer	37	No
SFTPA2 **(NM_001098668), AD**							
19GM00384	c.542A>G p.(Tyr181Cys) +/-	P		(3)	ILD, Cornelia de Lange syndrome	0.04	No
16GM00134	c.713G>C p.(Cys238Ser) +/-	P	(1)	(3)	ILD	39	No
17GM02170	c.713G>C p.(Cys238Ser) +/-	P	(1)	(3)	PF transplanted	41	No
17GM02726	c.713G>C p.(Cys238Ser) +/-	P	(1)	(3)	Fibrosing ILD	40	No
18GM00463	c.532G>A p.(Val178Met) +/-	P	(1,3)	(3)	PF, vasculitis	34	Yes (lung cancer in an uncle)
19GM00025	c.697T>A p.(Trp233Arg) +/-	P	(1,3)	(3)	PF, lepidic lung cancer	34	Yes (lepidic lung cancer and ILD in a brother, lung cancer in the father, PF in the mother)
19GM00503	c.532G>A p.(Val178Met) +/-	P	(1)	(3)	ILD	47	Yes (PF in the mother and lung cancer in a brother)
19GM01399	c.697T>A p.(Trp233Arg) +/-	P		(3)	ILD	42	Yes (lung cancer and PF in the father)
8822GM000939	c.635_654del p.(Tyr212Serfs*17) +/-	LP			PF	44	No
8823GM000322	c.623A>G p.(Tyr208Cys) +/-	LP			PF	33	Yes (lung cancer)

8823GM000914	c.725G>A p.(Arg242Gln) +/-	P		(3)	PF	NA	Yes (undefined)
18GM00397	c.532G>A p.(Val178Met) +/-	P	(3)	(3)	Fibrosing ILD, lepidic lung cancer	43	No
8822GM000844	c.713G>C p.(Cys238Ser) +/-	P		(3)	ILD and lung cancer	NA	Yes (ILD in the father)
***SFTPB* (NM_000542.5), AR**							
17GM02316	c.75G>A p.(Trp25*) / c.361delinsGAA p.(Pro121Glufs*95)	LP P	(4)	(5)	RDS at full term, congenital alveolar dysplasia	0	Yes (RDS in one sister)
***SFTPC* (NM_003018), AD**							
18GM00108	c.218T>C p.(Ile73Thr) +/-	P		(6)	PF	1	Yes (Lung cancer in the father)
18GM00398	c.203T>A p.(Val68Asp) +/-	LP			Fibrosing ILD	50	NA
18GM00409	c.218T>C p.(Ile73Thr) +/-	P	(1)	(6)	ILD	0.5	Yes (ILD and lung cancer in the father)
8819GM001610	c.218T>C p.(Ile73Thr) +/-	P		(6)	ILD	32	NA
8820GM000076	c.474del p.(Ser159Leufs*27) +/-	LP	(7)	(7)	Fibrosing ILD	5	No
8822GM000310	c.338A>G p.(Tyr113Cys) +/-	LP			PF	39	Yes (PF in the father)
8823GM000137	c.218T>C p.(Ile73Thr) +/-	P		(6)	ILD	21	Yes (bronchiectasis in the mother, COPD in the father)
8823GM000925	c.581T>C p.(Leu194Pro) +/-	LP		(8)	Fibrosing ILD	33	Yes (pulmonary aspergillosis in the mother with death at 30, PF and rheumatoid arthritis in the maternal grandfather)
8821GM000331	c.470del p.(Pro157Leufs*29) +/-	LP			RDS	0	NA, confirmed *de novo* status
8822GM000417	c.563T>C p.(Leu188Pro) +/-	LP		(8)	RDS at full term, ILD	0	No, confirmed *de novo* status
***ABCA3* (NM_001089), AR**							
17GM00786	c.2414+1G>C p. ? / c.875A>T p.(Glu292Val)	LP LP	(7)	(9)	Fibrosing ILD	43	No
18GM00548	c.3518C>G p.(Thr1173Arg) +/+	LP	(10)	(10)	ILD	2	Yes (ILD in a brother and in a paternal cousin)
18GM00673	c.4483_4507del p.(Val1495Cysfs*21) / c.875A>T p.(Glu292Val)	P LP	(1,7)	(9)	Fibrosing ILD	20	No

18GM01114	c.127C>T p.(Arg43Cys) /<br>c.3004G>A p.(Gly1002Ser)	LP<br>LP	(7)	(11)	PF	35	No
19GM00735	c.347T>C p.(Phe116Cys) /<br>c.838C>T p.(Arg280Cys)	LP<br>P	(7)	(12)	PF	20	Yes (Lung cancer in the maternal grandmother, PF in a maternal cousin)
8821GM000994	c.4237G>A p.(Gly1413Ser) /<br>c.4444C>T p.(Arg1482Trp)	LP<br>LP	(1,7,13)	(12)	ILD	30	No
8822GM000243	c.316C>T p.(Arg106*) /<br>c.875A>T p.(Glu292Val)	P<br>LP		(14)<br>(9)	ILD	3	No
8821GM001281	c.622C>T p.(Arg208Trp) +/+	P		(12)	Kystic emphysema	31	No
19GM00649	c.2068G>A p.(Glu690Lys) /<br>c.4615G>A p.(Asp1539Asn)	P<br>LP		(9)	RDS at full term, ILD and PHT	0	NA
19GM01428	c.743C>T p.(Pro248Leu) /<br>c.1755del p.(Thr586Profs*36)	LP<br>LP		(12)	RDS at full term	0	No
8822GM000212	c.737C>T p.(Pro246Leu) +/+	P		(15)	RDS at full term	0	NA
8823GM000892	c.3997_3998del p.(Arg1333Glyfs*24) +/+	P		(12)	RDS at full term, PHT	0	No
***NKX2-1* (NM_001079668), AD**							
19GM00219	c.1161C>A p.(Tyr387*) +/-	LP			ILD	3	NA, confirmed *de novo* status
18GM00926	c.1120del p.(Val374Tyrfs*7) +/-	LP			Fibrosing ILD	43	Yes (emphysema in the mother and in one brother, lung cancer in one brother)
18GM01458	c.949C>T p.(Gln317*) +/-	LP	(1)		Fibrosing ILD, hypothyroidy, rheumatic arthritis	36	No
8822GM000248	c.463+4A>G p.? +/-	P		(16)	PF, nail dystrophy, hypothyroidy	40	Yes (PF in the mother)
8822GM000916	c.583C>T p.(Arg195Trp) +/-	P		(17)	Fibrosing ILD, hypothyroidy, finger clubbing	2	No, confirmed *de novo* status
8820GM000593	c.973C>T p.(Gln325*) +/-	LP			Post-infection RDS, hypothyroidy	0	NA, confirmed *de novo* status
19GM01101	c.731A>G p.(Tyr244Cys) +/-	LP	(18)	(18)	RDS at full term, PHT, acinar dysplasia, hypothyroidy	0	No

***MARS1* (NM_004990), AR**							
8823GM000142	c.1700C>T p.(Ser567Leu) +/+	P		(19)	ILD, suspicion of PAP, preterm birth (30 weeks of gestation)	<1	NA
18GM00039	c.277C>T p.(Gln93*) /<br>c.1727C>T p.(Thr576Ile)	LP<br>LP			PAP	0	No
18GM00671	c.888G>C p.(Arg296Ser) /<br>c.1505G>A p.(Gly502Glu)	LP<br>LP			PAP	0.9	NA
19GM01251	c.854T>C p.(Ile285Thr) /<br>c.2114dup p.(Leu705Phefs*19)	LP<br>LP		(20)	RDS, PAP, cholestasis	0.0	No
8820GM000200	c.1700C>T p.(Ser567Leu) +/+	P		(19)	RDS, PAP	0	NA
8820GM000668	c.1700C>T p.(Ser567Leu) +/+	P		(19)	PAP	0	No
8823GM001074	c.1700C>T p.(Ser567Leu) +/+	P		(19)	PAP	0.16	NA
***CSF2RB* (NM_000395), AR**							
8822GM000756	c.631C>T p.(Arg211*) +/+	LP	(21)	(21)	PAP, PF	22	No
***FARSA* (NM_004461), AR**							
17GM01487	c.883C>T p.(Arg295Trp) +/+	LP	(22)	(22)	ILD, hemosiderosis, hypotonia, developmental delay	<1	Yes (death at 1 month in one brother, 2 miscarriages in the mother)
***STING1* (NM_198282), AD**							
17GM01795	c.463G>A p.(Val155Met) +/-	P		(23)	Fibrosing ILD, vasculitis	7	No
8821GM000541	c.841C>T p.(Arg281Trp) +/-	P		(24)	ILD	11	No
***COPA* (NM_004371), AD**							
17GM01384	c.698G>A p.(Arg233His) +/-	P		(25)	ILD, joint involvement	7	No
***TBX4* (NM_001321120), AD**							
8823GM000063	c.1200del p.(Glu401Argfs*16) +/-	LP			RDS at full term, ILD, PHT	0.41	Yes (lung dysplasia, death in the first hours of life in one paternal aunt)

Abbreviations: AD: autosomal dominant; AR: autosomal recessive; +/-: heterozygous; +/+: homozygous; ACMG: American College of Medical Genetics and Genomics; LP: likely pathogenic; P: pathogenic; PF: pulmonary fibrosis, ILD: interstitial lung disease; RDS: respiratory distress syndrome, PHT: pulmonary hypertension, PAP: pulmonary alveolar proteinosis; COPD: chronic obstructive pulmonary disease, NA: not available

References

1. Brudon A, Legendre M, Mageau A, Bermudez J, Bonniaud P, Bouvry D, et al. High risk of lung cancer in surfactant-related gene variant carriers. Eur Respir J. mai 2024;63(5):2301809.

2. Nathan N, Giraud V, Picard C, Nunes H, Dastot-Le Moal F, Copin B, et al. Germline SFTPA1 mutation in familial idiopathic interstitial pneumonia and lung cancer. Hum Mol Genet. 15 avr 2016;25(8):1457-67.

3. Legendre M, Butt A, Borie R, Debray MP, Bouvry D, Filhol-Blin E, et al. Functional assessment and phenotypic heterogeneity of SFTPA1 and SFTPA2 mutations in interstitial lung diseases and lung cancer. Eur Respir J. déc 2020;56(6):2002806.

4. Fleury M, Delestrain C, Roditis L, Perisson C, Renoux MC, Thumerelle C, et al. Surfactant protein B deficiency: the RespiRare cohort. Thorax. 17 janv 2025;80(2):109-12.

5. Nogee LM, Garnier G, Dietz HC, Singer L, Murphy AM, deMello DE, et al. A mutation in the surfactant protein B gene responsible for fatal neonatal respiratory disease in multiple kindreds. J Clin Invest. avr 1994;93(4):1860-3.

6. Nogee LM, Dunbar AE, Wert S, Askin F, Hamvas A, Whitsett JA. Mutations in the surfactant protein C gene associated with interstitial lung disease. Chest. mars 2002;121(3 Suppl):20S-21S.

7. Diesler R, Legendre M, Si-Mohamed S, Brillet PY, Wemeau L, Manali ED, et al. Similarities and differences of interstitial lung disease associated with pathogenic variants in SFTPC and ABCA3 in adults. Respirology. avr 2024;29(4):312-23.

8. Guillot L, Epaud R, Thouvenin G, Jonard L, Mohsni A, Couderc R, et al. New surfactant protein C gene mutations associated with diffuse lung disease. J Med Genet. juill 2009;46(7):490-4.

9. Bullard JE, Wert SE, Whitsett JA, Dean M, Nogee LM. ABCA3 mutations associated with pediatric interstitial lung disease. Am J Respir Crit Care Med. 15 oct 2005;172(8):1026-31.

10. Flamein F, Riffault L, Muselet-Charlier C, Pernelle J, Feldmann D, Jonard L, et al. Molecular and cellular characteristics of ABCA3 mutations associated with diffuse parenchymal lung diseases in children. Hum Mol Genet. 15 févr 2012;21(4):765-75.

11. Agrawal A, Hamvas A, Cole FS, Wambach JA, Wegner D, Coghill C, et al. An intronic ABCA3 mutation that is responsible for respiratory disease. Pediatr Res. juin 2012;71(6):633-7.

12. Somaschini M, Nogee LM, Sassi I, Danhaive O, Presi S, Boldrini R, et al. Unexplained neonatal respiratory distress due to congenital surfactant deficiency. J Pediatr. juin 2007;150(6):649-53, 653.e1.

13. Le Brun M, Nathan N, Louvrier C, Legendre M, Feuillet S, Frija-Masson J, et al. Efficacy and safety of CFTR modulators in patients with interstitial lung disease caused by ABCA3 transporter deficiency. ERJ Open Res. mars 2025;11(2):00701-2024.

14. Shulenin S, Nogee LM, Annilo T, Wert SE, Whitsett JA, Dean M. ABCA3 gene mutations in newborns with fatal surfactant deficiency. N Engl J Med. 25 mars 2004;350(13):1296-303.

15. Jouza M, Jimramovsky T, Sloukova E, Pecl J, Seehofnerova A, Jezova M, et al. A Newly Observed Mutation of the ABCA3 Gene Causing Lethal Respiratory Failure of a Full-Term Newborn: A Case Report. Front Genet. 2020;11:568303.

16. Thorwarth A, Schnittert-Hübener S, Schrumpf P, Müller I, Jyrch S, Dame C, et al. Comprehensive genotyping and clinical characterisation reveal 27 novel NKX2-1 mutations and expand the phenotypic spectrum. J Med Genet. juin 2014;51(6):375-87.

17. Guillot L, Carré A, Szinnai G, Castanet M, Tron E, Jaubert F, et al. NKX2-1 mutations leading to surfactant protein promoter dysregulation cause interstitial lung disease in « Brain-Lung-Thyroid Syndrome ». Hum Mutat. févr 2010;31(2):E1146-1162.

18. Soreze Y, Nathan N, Jegard J, Hervieux E, Clermidi P, Sileo C, et al. Acinar Dysplasia in a Full-Term Newborn with a NKX2.1 Variant. Neonatology. 2024;121(1):133-6.

19. Hadchouel A, Wieland T, Griese M, Baruffini E, Lorenz-Depiereux B, Enaud L, et al. Biallelic Mutations of Methionyl-tRNA Synthetase Cause a Specific Type of Pulmonary Alveolar Proteinosis Prevalent on Réunion Island. Am J Hum Genet. 7 mai 2015;96(5):826-31.

20. Alzaid M, Alshamrani A, Al Harbi AS, Alenzi A, Mohamed S. Methionyl-tRNA synthetase novel mutation causes pulmonary alveolar proteinosis. Saudi Med J. févr 2019;40(2):195-8.

21. Papiris SA, Louvrier C, Fabre A, Kaklamanis L, Tsangaris I, Frantzeskaki F, et al. CSF2RB mutation-related hereditary pulmonary alveolar proteinosis: the « long and winding road » into adulthood. ERJ Open Res. nov 2023;9(6):00703-2023.

22. Charbit-Henrion F, Goguyer-Deschaumes R, Borensztajn K, Mirande M, Berthelet J, Rodrigues-Lima F, et al. Systemic inflammatory syndrome in children with FARSA deficiency. Clin Genet. mai 2022;101(5-6):552-8.

23. Liu Y, Jesus AA, Marrero B, Yang D, Ramsey SE, Sanchez GAM, et al. Activated STING in a vascular and pulmonary syndrome. N Engl J Med. 7 août 2014;371(6):507-18.

24. Lin B, Berard R, Al Rasheed A, Aladba B, Kranzusch PJ, Henderlight M, et al. A novel STING1 variant causes a recessive form of STING-associated vasculopathy with onset in infancy (SAVI). J Allergy Clin Immunol. nov 2020;146(5):1204-1208.e6.

25. Watkin LB, Jessen B, Wiszniewski W, Vece TJ, Jan M, Sha Y, et al. COPA mutations impair ER-Golgi transport and cause hereditary autoimmune-mediated lung disease and arthritis. Nat Genet. juin 2015;47(6):654-60.
